# Mapping *Blastocystis* epidemiology and diagnostics across Europe: A One Health survey of professional practice, perceptions and unmet needs

**DOI:** 10.1016/j.onehlt.2026.101603

**Published:** 2026-10-01

**Authors:** Eylem Akdur-Öztürk, Ana M. Figueiredo, Isabel Guadano-Procesi, Ayşe Övgü Kinay, Erdoğan Malatyali, Esma Kozan, Ferhat Matur, Anja Godfrey, WG1 consortium, Eleni Gentekaki, Anastasios D. Tsaousis, David Carmena, Funda Dogruman-Al

**Affiliations:** a Department of Parasitology, Faculty of Medicine, Çukurova University, Adana, Türkiye; b Department of Biology and CESAM, University of Aveiro, Aveiro, Portugal; c Department of Clinical Sciences and Translational Medicine, Faculty of Medicine, University of Tor Vergata, Rome, Italy; d Department of Computer Science, Faculty of Science, Dokuz Eylul University, İzmir, Türkiye; e Department of Parasitology, Faculty of Medicine, Aydın Adnan Menderes University, Aydın, Türkiye; f Department of Parasitology, Faculty of Veterinary Medicine, Afyon Kocatepe University, Afyonkarahisar, Türkiye; g Department of Biology, Faculty of Science, Dokuz Eylul University, Izmir, Türkiye; h University of Kent, School of Natural Sciences, Canterbury, UK; i Department of Veterinary Medicine, University of Nicosia School of Veterinary Medicine, Nicosia, Cyprus; j Laboratory of Molecular and Evolutionary Parasitology, School of Natural Sciences, University of Kent, Canterbury, Kent, UK; k Parasitology Reference and Research Laboratory, Spanish National Centre for Microbiology, Health Institute Carlos III, Majadahonda, Spain; l Center for Biomedical Research Network (CIBER) in Infectious Diseases, Health Institute Carlos III, Madrid, Spain; m Division of Medical Parasitology, Department of Medical Microbiology, Faculty of Medicine, Gazi University, Ankara, Türkiye

**Keywords:** *Blastocystis*, Diagnosis, Subtyping, Epidemiology, Standardisation, One Health

## Abstract

*Blastocystis* is one of the most prevalent intestinal protists in humans and animals, yet its clinical significance remains unresolved, and its diagnosis is poorly standardised. We aimed to map professional practice, perceptions, and unmet needs in *Blastocystis* epidemiology and diagnostics across Europe. Within the European Cooperation in Science and Technology (COST) Action CA21105, Working Group 1 conducted a multinational cross-sectional survey using a dedicated structured online questionnaire distributed through national representatives and European professional networks across COST member countries, and responses on institutional profiles, diagnostic and subtyping workflows, perceived clinical relevance, and treatment were analysed using Pearson chi-square tests. A total of 439 fully completed, valid questionnaires from 38 countries were evaluated. Human-derived stool samples predominated (75.3%). Microscopy remained the principal screening method (61.6%), whereas only 16.9% used molecular methods alone, and 75.0% performed no subtyping. Subtyping was associated with institution type and research purpose (*p* < 0.001): universities and epidemiological laboratories drove barcoding/Sanger sequencing, whereas health institutions most often did not subtype. Reporting to clinicians was inconsistent (20.5% never reported). Most clinicians (96.2%) linked *Blastocystis* to gastrointestinal symptoms, and metronidazole was the predominant treatment choice (57.8%) despite inconsistent efficacy. Respondents endorsed standardisation and data-sharing, citing the lack of guidelines and inter-group communication (44.6%), as well as insufficient funding, as the principal barriers to improving practices. Overall, a marked gap separates near-universal professional conviction in the clinical relevance of *Blastocystis* from the diagnostic and molecular capacity to test it; these findings provide an evidence base for uniform, One Health–oriented guidelines for *Blastocystis* diagnosis and surveillance across Europe.

## Highlights

• Largest multinational European survey of *Blastocystis* diagnostics (439 replies, 38 countries).
• Microscopy still dominates (61.6%); 75% of laboratories perform no subtyping.
• Reporting of *Blastocystis* to clinicians is inconsistent and often undefined.
• Metronidazole predominates (57.8%) despite weak efficacy and microbiome harm.
• Main barrier is lack of guidelines and coordination, not funding.

## 1 Introduction

*Blastocystis* is a ubiquitous, genetically diverse stramenopile and represents one of the most prevalent intestinal protists identified in human and animal populations globally [1], [2], [3]. As a zoonotic organism, it is widely distributed among numerous animal species, which serve as reservoirs for human colonisation [4], [5], [6]. Despite its high prevalence, its fundamental biological roles remain a subject of intense investigation. *Blastocystis* is increasingly regarded not merely as a parasite, but as a constituent of the healthy intestinal microbiota [4], [7], [8].

The clinical importance of *Blastocystis* remains unresolved, as its pathogenic role is considered only potential and not definitively established. This uncertainty stems from the fact that it is frequently detected in asymptomatic individuals with high microbial alpha-diversity, leading to a “pathogenicity paradox.” It remains unclear whether the organism exerts a direct modulatory effect on gut microbial ecology, potentially acting as an immunomodulatory symbiont, or whether it preferentially colonises hosts who already possess a stable and diverse microbiome [4], [9], [10]. Consequently, modern research perspectives are increasingly moving away from regarding *Blastocystis* as a parasite, considering instead that its presence may reflect a state of eubiosis rather than drive disease [11], [12].

The lack of uniform diagnostic methodologies and standardised reporting frameworks, together with the still-incomplete understanding of *Blastocystis* epidemiology, particularly regarding animal reservoirs and environmental sources, substantially constrains an accurate and comprehensive evaluation of its public health significance [7], [13], [14]. This methodological heterogeneity and epidemiological uncertainty hinder reliable prevalence estimates, limit cross-study comparability, and ultimately impede a coherent understanding of the organism's distribution, host range, along with public-health and veterinary relevance [15], [16]. Moreover, in many routine diagnostic settings, standardised procedures for the systematic detection of *Blastocystis* are not implemented, resulting in substantial underestimation of its prevalence and potential misclassification of its occurrence [16], [17].

To address these knowledge gaps, the European Cooperation in Science and Technology (COST) Action CA21105 established Working Group 1 (WG1) with the primary objective of conducting the first systematic, European-wide assessment of *Blastocystis* epidemiology and current diagnostic methodologies [18]. Within this strategic framework, WG1 developed and implemented an online survey, titled “Mapping *Blastocystis* Epidemiology and Diagnostics”, designed to evaluate the current landscape of clinical and laboratory practices. This questionnaire was specifically developed to systematically evaluate the level of professional awareness and perception of *Blastocystis* among clinicians, veterinarians, and microbiologists across Europe. In addition, it aimed to comprehensively characterise the diagnostic workflows and, where applicable, the subtyping strategies currently adopted in clinical, veterinary, and environmental settings, with particular attention to methodological variability and operational standards [18].

Here, we present and analyse the data generated through this survey. These findings are intended to inform the development of uniform, evidence-based guidelines and standardised technical protocols. By advocating for methodological standardisation across institutions, this study provides evidence to support subsequent initiatives to strengthen inter-laboratory comparability, improve the reproducibility and robustness of *Blastocystis* epidemiological data, and facilitate systematic knowledge exchange within the European scientific community.

## 2 Materials and methods

### 2.1 Study design

The methodology for this study was developed by Working Group 1 (WG1), “Mapping of *Blastocystis* Epidemiology and Diagnostics,” of the COST Action CA21105, which implemented an online survey to map *Blastocystis* diagnostic and epidemiological practices across Europe. The survey process followed a comprehensive workflow involving country selection, institution estimation, survey design, pilot testing, recruitment, and systematic data collection and analysis (Fig. 1). To ensure representative coverage, the study targeted COST full-member countries, where National Representatives (NRs) identified and recruited participants from the human, veterinary, and environmental sectors through formal invitations and QR codes. Eligible participants were professionals actively involved in the diagnosis, surveillance, or research of *Blastocystis* in the human, veterinary, or environmental sectors within COST member countries; incomplete or duplicate submissions and responses from individuals with no professional involvement with *Blastocystis* or working outside the European Union/neighbouring countries were excluded. In addition to recruitment through NRs, the survey was disseminated via European professional networks, including the European Society of Clinical Microbiology and Infectious Diseases (ESCMID) and its affiliated study groups, to broaden participation across clinical, veterinary, and environmental disciplines [18]. The final questionnaire, hosted on Google Forms, was organised into three specialised sections covering institutional profiles, awareness and clinical management, and detailed laboratory methodologies for detection and subtyping (Supplementary File 1). Prior to full deployment, the questionnaire was validated through a pilot study conducted in Türkiye. The pilot assessed item clarity, completeness, internal consistency, and the time required for completion; A total of 101 respondents participated, and the issues identified (e.g., ambiguous item wording and redundant response options) informed targeted revisions before the instrument was finalised and further refined by a multidisciplinary panel of experts to ensure technical accuracy and the elimination of ambiguous interpretations [18]. A glossary of key terms and definitions used in the final version of the questionnaire can be seen in Supplementary File 2.

**Fig. 1:**
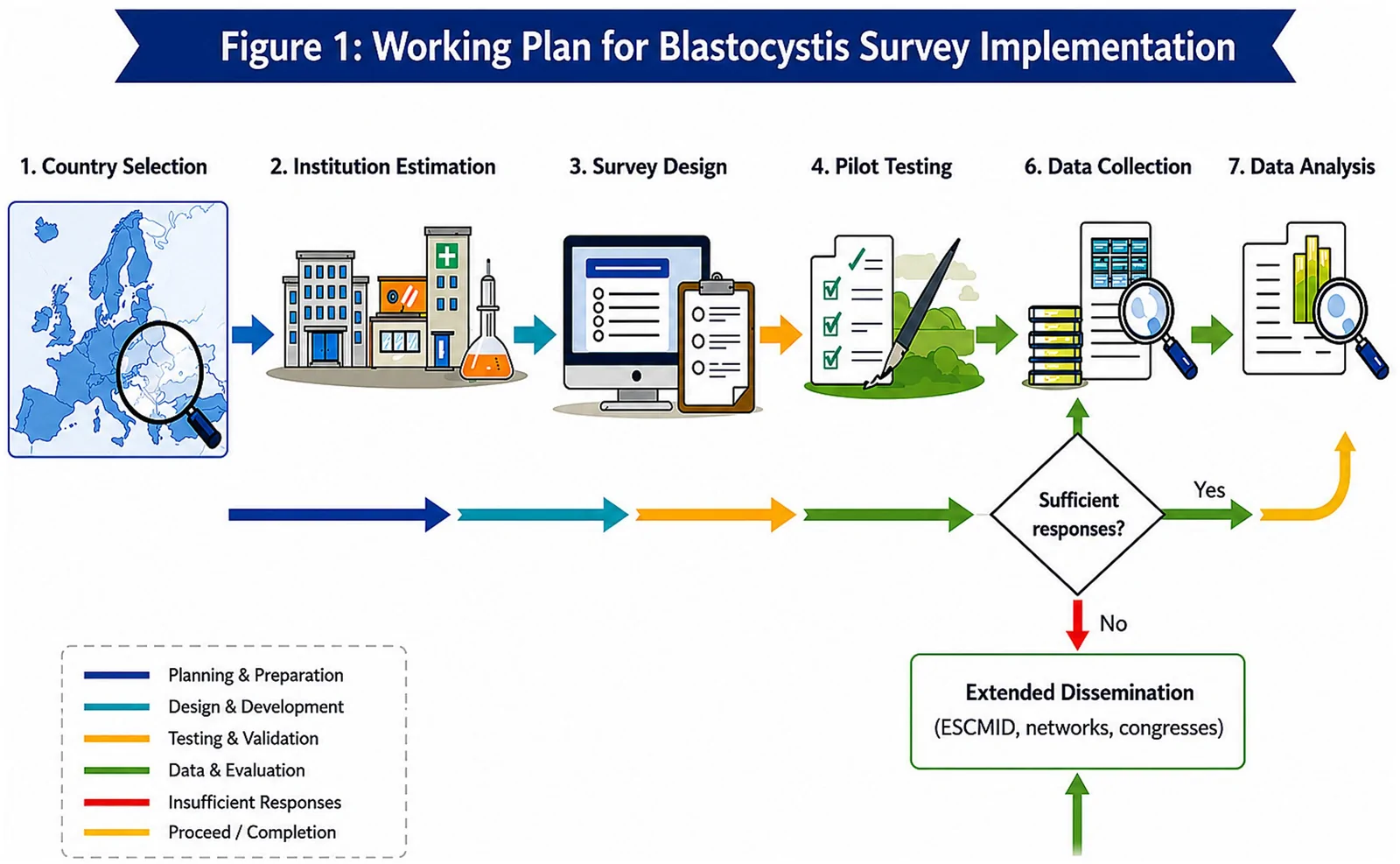
Working plan for *Blastocystis* survey implementation. The workflow comprised country selection, institution estimation, survey design, pilot testing, recruitment, data collection, and data analysis, followed by dissemination through the COST Action CA21105 network.

### 2.2 Statistical analysis

All statistical analyses were performed using IBM SPSS Statistics for Windows, Version 30.0 (IBM Corp., Armonk, NY, USA). Findings related to categorical variables were summarised using frequency (*n*) and percentage (%) values; the general distribution of the data was visualised using bar charts for multiple-choice questions and pie charts for questions with a small number of mutually exclusive response options. Prior to cross-tabulation analyses, a specific data-cleaning protocol was applied to enhance statistical reliability. To prevent attribution bias in comparative analyses and to ensure the independence assumption of the groups, participants who selected more than one category in the relevant questions (e.g.*,* those who marked both university and health institution as the type of institution) were excluded, creating completely independent “pure cohorts.” Questions that, by their nature, allowed multiple selections were defined as “multiple-response sets”, and each preference was calculated separately within the independent cohorts. Furthermore, to prevent violations of the expected cell-count assumptions, a fundamental requirement of chi-square analyses, “Not Answered (NA)” cases, which could reduce statistical power, and “Other” categories with insufficient frequency were excluded. Because some row variables were multiple-response sets, in which a single respondent could contribute to more than one category, the omnibus chi-square statistic of these tables was interpreted as a descriptive summary of the overall association, and inference was based on the pairwise comparisons of column proportions for each category. When more than 20% of cells had expected counts below five, the relevant comparisons were confirmed with Fisher's exact test (Fisher–Freeman–Halton test for tables larger than 2 × 2). For the comparison between institutional focus and the use of OMICs approaches, institutions reporting a single additional, non-overlapping area (e.g., human health and food, water and environmental testing) were retained under their primary health domain, whereas those reporting both a human and an animal health focus were excluded. Finally, to assess whether the exclusion of respondents with multiple selections introduced bias, each excluded group was compared with the corresponding pure cohort on the same key variables (Supplementary Table 1).

The Pearson chi-square test of independence was used to evaluate the relationships between independent groups (institution types, primary purpose of the laboratory, sample sources, etc.) and the preferred screening/subtyping methods and treatment approaches. Where statistically significant relationships were identified, post-hoc comparisons of column proportions with Bonferroni correction were applied to determine which specific groups accounted for the difference. In all evaluations, the significance level was set at *p* < 0.05.

## 3 Results

### 3.1 Respondents and institutional profile

A total of 439 valid questionnaires were analysed, encompassing responses from 38 European countries. Respondents were mainly from Türkiye (23.0%), followed by Italy (17.1%), Spain (13.7%), and France (6.2%); the remaining countries accounted for 40.1% (Fig. 2; Supplementary Table 2). No responses were obtained from Armenia, Georgia, Luxembourg, and the Republic of Moldova.

**Fig. 2:**
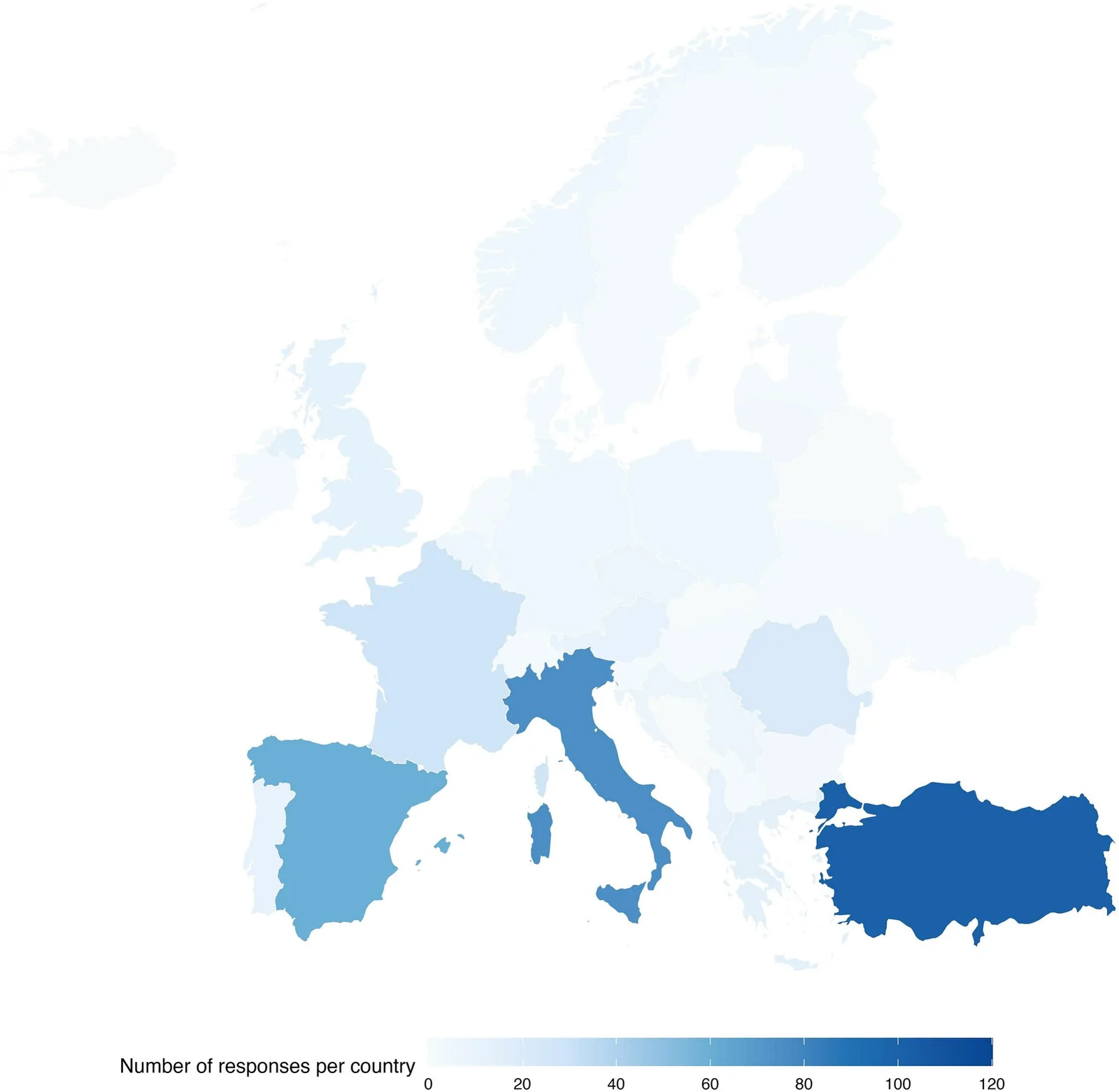
Map representation of the number of valid responses per European country assessed in this study. Colour intensity indicates the number of responses per country (range 0–120).

Most respondents were employed in health institutions (46.7%) and universities (44.2%), followed by research institutes (7.0%). The remaining respondents worked in veterinary clinics/diagnostic laboratories (1.4%), government institutions (0.9%), or the pharmaceutical sector (0.2%). Most respondents conducted microbiological (38.9%) and clinical (35.0%) work on *Blastocystis*, with fewer working in epidemiology (12.8%) or combined approaches (12.3%). The remainder focused on molecular biology, education, environmental risk and public health, and food safety (1%).

A significant association was observed between institution type and the primary purpose of *Blastocystis*-related activities (χ^2^ = 73.779, *p* < 0.001; *n* = 316). Post-hoc tests revealed that health institutions were significantly more likely to focus on clinical applications (*p* < 0.05), whereas universities showed greater engagement in epidemiological research (*p* < 0.05). In contrast, both types of institutions demonstrated a comparable level of involvement in microbiological investigations.

Most participating institutions were primarily focused on human health (73.4%), followed by animal health (18.0%) and environmental/One Health activities (8.6%). The vast majority did not conduct OMICs studies (90.2%). Among the minority that did (9.8%), genomics was the most commonly used approach (40.0%), followed by combined multi-OMICs strategies (22.5%), metagenomics (17.5%), and microbiome studies (12.5%). Transcriptomics (5.0%) and proteomics (2.5%) were used less frequently. After excluding institutions that reported both a human and an animal health focus (*n* = 36), the use of OMICs approaches did not differ between institutions with an exclusive or predominant focus on human health and those focused on animal health (7.3% vs. 7.1%; χ^2^ = 0.003, *p* = 0.957; *n* = 369). In contrast, institutions with a combined human and animal health focus reported conducting OMICs studies more frequently than the remaining institutions (33.3% vs. 7.3%; Fisher's exact test, *p* < 0.001) (Supplementary Table 1). However, given the small size of this subgroup and the fact that the factors underlying this difference were not explored in the present survey, this finding should be regarded as exploratory and warrants further investigation.

Overall, 79 respondents reported more than one institution type, 51 reported more than one purpose for their *Blastocystis* work, 36 analysed samples from more than one source, and 27 clinicians reported combined treatment modalities; these respondents were excluded from the corresponding pure-cohort comparisons. When compared with the pure cohorts on the same key variables (Supplementary Table 1), the excluded groups showed no significant differences in sample-preservation methods, or treatment criteria, and respondents with multiple institutional affiliations did not differ in subtyping methods. However, respondents with multiple institutional affiliations more often reported a clinical purpose (58.1% vs. 42.4%) and the use of real-time PCR (31.0% vs. 19.6%), and less often reported never communicating *Blastocystis* findings to clinicians (10.0% vs. 21.8%); respondents with multiple purposes more often used trichrome staining, concentration methods, culture for screening, and barcoding/Sanger sequencing for subtyping (34.0% vs. 10.6%); laboratories analysing samples from multiple sources more often used culture and conventional PCR (all *p* < 0.05).

### 3.2 Routine diagnostic practices

Human-derived stool samples were by far the most common biological specimen analysed, reported by 75.3% of respondents, followed by animal-derived stool samples (15.6%). Environmental matrices reported by respondents (chiefly water, soil, and fresh produce), together with samples of multiple origins, were examined much less frequently, accounting for only 0.7–3.0% of responses each. For diagnostic purposes, most respondents processed and analysed fewer than 100 *Blastocystis* samples annually, regardless of sample origin: 37.3% for human samples, 60.8% for animal samples, and 70.9% for environmental samples. In contrast, high-throughput testing (> 5000 samples per year) was relatively uncommon, reported by only 9.2% of laboratories analysing human samples, 3.0% examining animal samples, and 6.3% evaluating environmental samples (Table 1). The following countries reported testing more than 5000 samples per year: Albania, Austria, Bosnia and Herzegovina, Croatia, France, Germany, Italy, Serbia, Spain, and Türkiye. Among respondents working with human-derived samples, 62.8% routinely reported *Blastocystis* findings to clinicians. By comparison, 20.5% never reported such findings, while 16.7% did so only under specific circumstances; in the latter group, reporting was most commonly prompted by a significant *Blastocystis* burden (defined by the presence of ≥5 cells in each microscopic field, 48.8%), the presence of compatible clinical symptoms (31.7%), or a specific request from the attending clinician (19.5%). However, the lack of standardised criteria for defining parasite burden, highlights the need to establish objective and harmonised reporting thresholds, ideally incorporating quantitative molecular markers, to improve consistency across laboratories and clinical settings.

**Table 1:** Percentage of analysed samples per year for respondents diagnosing *Blastocystis* in human-, animal-, and environmental-origin samples.

Number of samples per year	Human-origin (%)	Animal-origin (%)	Environmental-origin (%)
< 100	37.3	60.8	70.9
100–499	16.4	19.2	15.2
500–999	17.4	8.5	7.6
1000–5000	19.7	8.5	–
> 5000	9.2	3.0	6.3

Regarding stool-sample preservation for molecular testing, the most used methods were freezing at −20 °C (26.0%), refrigeration at 4 °C (19.5%), and freezing at −80 °C (18.6%). Additional approaches included storage in 70% ethanol (8.8%) or commercial preservation devices/solutions (5.1%). Among the latter, the systems most frequently mentioned were the Cary-Blair medium, the Copan store device, DNA/RNA Shield, and DNA/RNAlater (each 20.0%), followed by the Roche STAR buffer and a Zymo buffer (each 10.0%). Notably, 22.0% of respondents reported not using any preservation method, as they worked only with fresh faecal material.

For *Blastocystis* screening, microscopy remained the most widely used diagnostic approach, employed by 61.6% of respondents, while 16.6% relied on molecular methods and 21.5% on a combination of both (Fig. 3). Among respondents using microscopy, 59.4% examined samples directly without enrichment. Among those incorporating an enrichment step (40.6%), cell-concentration methods were by far the most commonly used (86.0%), whereas in vitro culture alone (7.0%) or a combination with concentration methods (7.0%) were much less frequently employed. For microscopic detection, Lugol's iodine was the most used preparation method (24.3%), followed by saline wet mounts (8.5%) and trichrome staining (3.7%). Despite this, the combined use of multiple preparation and staining methods was the most frequent overall strategy, reported by 36.5% of respondents (Fig. 3).

**Fig. 3:**
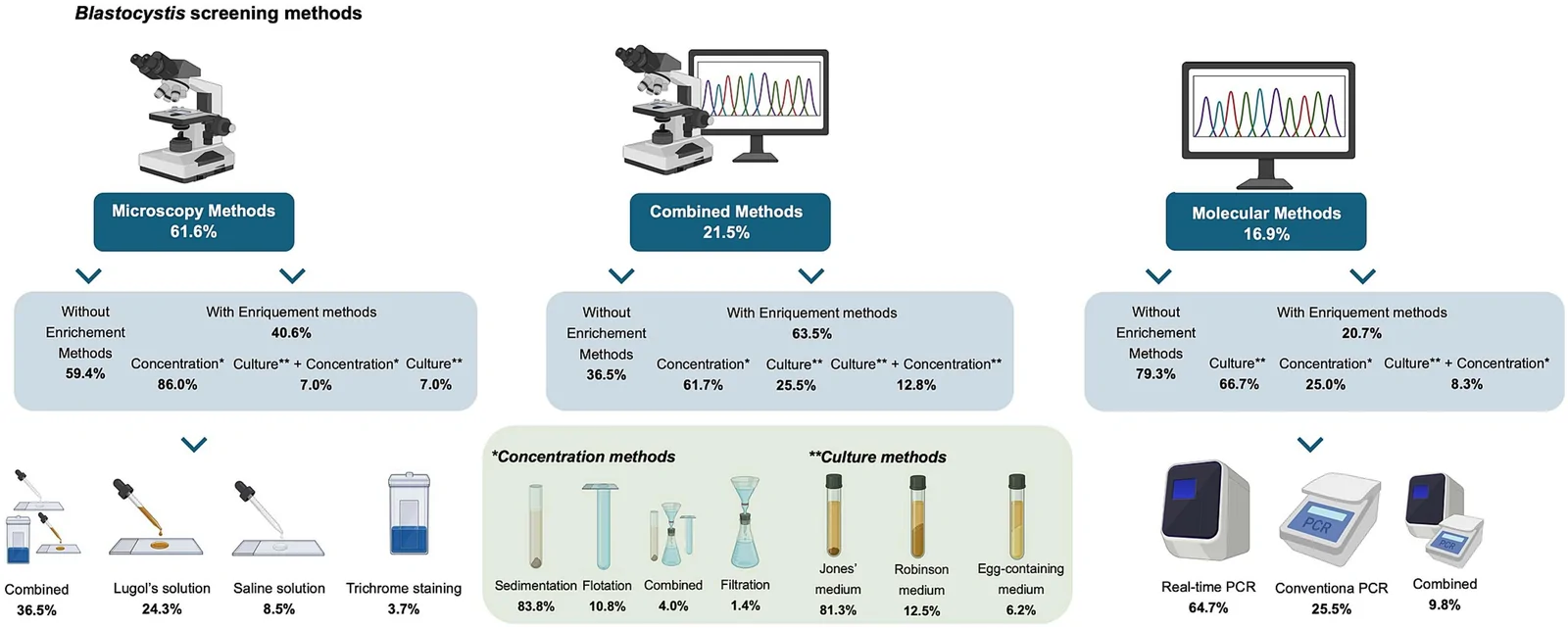
*Blastocystis* screening methods and corresponding percentages reported by respondents, showing the relative use of microscopy, combined, and molecular approaches, together with associated enrichment, concentration, culture, staining, and PCR options.

Among respondents using molecular methods for *Blastocystis* screening, most analysed samples directly without prior enrichment (79.3%). Of the minority that incorporated an enrichment step (20.7%), in vitro culture was the preferred approach (66.7%), followed by cell-concentration methods (25.0%) or a combination of both techniques (8.3%). Real-time PCR (RT-PCR) was the most frequently employed molecular method (64.7%), followed by conventional PCR (25.5%) or a combination of both assays (9.8%) (Fig. 3). Among laboratories combining microscopy and molecular methods, the majority (63.5%) incorporated an enrichment step before analysis; in this group, parasite-concentration methods were the most used (61.7%), followed by in vitro culture (25.5%) and the combined use of both approaches (12.8%) (Fig. 3).

Among the enrichment methods reported, cell concentration by sedimentation was the most frequently used technique (83.8%), including formalin-ether sedimentation, Parasep® systems (Apacor; Wokingham, Berkshire, UK), and Bailenger-based kits. Flotation methods were used less frequently (10.8%), typically involving zinc sulphate or saturated salt/sugar solutions, while filtration (1.4%) and combined approaches (4.0%) were rarely adopted. For in vitro culture, Jones' medium was the predominant choice (81.3%), followed by Robinson medium (12.5%) and egg-containing medium (6.2%) (Fig. 3).

A significant association was observed between the primary purpose of *Blastocystis*-related activities and the methods used for screening *Blastocystis* (χ^2^ = 69.247, *p* < 0.001). Post-hoc tests indicated that laboratories conducting research for epidemiological purposes were significantly more likely to use conventional PCR compared to those in clinical or microbiological settings (*p* < 0.05). Conversely, laboratories focused on microbiological purposes showed a significantly higher use of microscopy-based methods than those operating strictly in clinical environments (*p* < 0.05).

Regarding DNA extraction and purification, most respondents (86.3%) relied on commercially available kits. A small proportion used in-house methods based on faeces or cultured supernatants (4.9% each), while combined approaches were uncommon (3.9%). Among commercial products, those from QIAGEN (Hilden, Germany) were most frequently reported (47.0%), including the QIAamp Fast DNA Stool Mini Kit, QIAamp PowerFecal Pro DNA Kit, and QIAamp PowerSoil DNA Isolation Kit. The STARMag™ S96H N kit from Seegene (Seoul, South Korea) was also used (11.0%). A diverse group of other commercial systems accounted for the remaining 42.0%, including the Hologic Novodiag® Stool Parasites Assay (Marlborough, MA, USA), Zymo Quick-DNA Fecal/Soil Microbe Miniprep Kit (Irvine, CA, USA), and Invitrogen PureLink™ Microbiome Genomic DNA Purification Kit (Carlsbad, CA, USA).

Among respondents using conventional PCR for *Blastocystis* diagnosis, the vast majority (92.0%) used the pan-*Blastocystis* primer set RD5 and BhRDr [31], while a small proportion (8.0%) used alternative primer sets such as Blast 505–532 and Blast 998–1017 [32] or ST-specific primers [33]. Among RT-PCR users, the most frequently reported assay was the Seegene Allplex™ GI-Parasite Assay (45.5%). Other commonly used approaches included the primer pair BL18SPPF1 and BL18SR2PP (15.2%) [34], the BioSynex (Strasbourg, France) Ampliquick® Protozoans Kit (9.1%), and the Hologic Novodiag® Stool Parasites Assay (6.0%). A further 24.2% of respondents reported using other commercial kits, including the Roche (Basel, Switzerland) LightMix Gastro Parasite Multiplex PCR Assay or combined approaches such as in-house TaqMan-based systems (Blasto-FWD F5 and Blasto R-F2 primer set plus probes) [35]. For sequence analysis, GenBank was the most frequently used platform (59.7%), followed by PubMLST (31.6%) and online resources from the London School of Hygiene & Tropical Medicine (8.7%).

### 3.3 Subtyping

Most respondents (75.0%) reported not performing *Blastocystis* subtyping in their laboratories. Among those that did, the most frequently used methods were barcoding combined with Sanger sequencing (8.2%), sequence-tagged-site PCR (6.6%), next-generation sequencing (NGS; 5.1%), and combined approaches (5.1%). Among laboratories using NGS for subtyping, the Illumina (San Diego, CA, USA) platform (MiSeq, NovaSeq, NextSeq) was the most commonly employed (83.3%), followed by Oxford Nanopore Technologies (Oxford, UK; 11.1%), while a small proportion (5.6%) used both platforms.

An analysis of the relationship between institution type (universities vs. health institutions) and subtyping methods revealed a significant association (χ^2^ = 73.191, *p* < 0.001; *n* = 301). Post-hoc analyses showed that universities were more likely to use barcoding/Sanger sequencing and STS-PCR approaches (*p* < 0.05), whereas health institutions were more likely to report not conducting subtyping (*p* < 0.05). No significant differences were observed between institution types in NGS use.

The analysis of the relationship between research purpose (clinical, epidemiological, or microbiological) and *Blastocystis* subtyping methods (NGS, barcoding and Sanger sequencing, STS-PCR, or no subtyping) showed a significant overall association (χ^2^ = 68.313, *p* < 0.001; *n* = 322). Post-hoc comparisons revealed marked differences in subtyping practices across laboratories, depending on their research purposes. Laboratories engaged in epidemiological studies were significantly more likely to use barcoding and Sanger sequencing for subtyping than those with a clinical or microbiological orientation (*p* < 0.05). Microbiological settings also used this approach more frequently than strictly clinical settings. Conversely, both clinical and microbiological laboratories reported a significantly greater tendency not to perform subtyping than epidemiological laboratories (*p* < 0.05).

When asked about the potential impact of standardised screening and subtyping methods for *Blastocystis* at the regional level, most respondents (60.4%) believed their implementation would be significant, particularly by improving reliability, comparability, and diagnostic consistency across regions. Additional perceived benefits included enhanced ability of clinicians to interpret *Blastocystis* findings and guide patient management (14.4%), as well as increased understanding of local prevalence patterns and subtype distribution (10.2%). Only a small minority (7.0%) considered that standardisation would have no impact, 5.9% considered it would have a moderate impact, and 2.1% were uncertain about its value.

At the national level, most respondents (55.7%) considered that standardised screening and subtyping methods would have a substantial impact by improving the reliability of results, enhancing diagnostic and surveillance accuracy, and strengthening national databases. A further 32.8% recognised their value for advancing understanding of *Blastocystis* epidemiology and pathogenicity, thereby supporting more informed clinical decision-making. Only 3.3% believed that standardisation would have no impact, 2.7% believed it would have a moderate impact, and 5.5% were uncertain.

At the international level, support for standardisation was even stronger. Most respondents (78.3%) emphasised its importance for harmonising screening and subtyping procedures across Europe, thereby enhancing the comparability of diagnostic, surveillance, and investigation data. An additional 13.3% highlighted its value in clarifying *Blastocystis* epidemiology and in better understanding the relationships between subtype distribution, symptomatology, and pathogenicity. Only 4.8% considered that standardisation would have no impact, and 3.6% were unsure of its potential benefits.

Respondents were asked to identify the main barriers to developing standardised methods for *Blastocystis* screening and subtyping in investigative and surveillance settings. The most frequently cited gap was the lack of detailed guidelines and effective communication among research groups (44.6%). Other important barriers included limited knowledge and awareness of *Blastocystis* (26.2%) and insufficient laboratory infrastructure and funding (15.4%). Only 13.8% were uncertain about key missing elements.

When asked about the practical challenges associated with *Blastocystis* screening and subtyping, respondents identified a range of difficulties. Selecting the most appropriate diagnostic or subtyping method was the most cited single challenge (21.3%), followed by access to suitable test samples (15.0%). Difficulties in obtaining relevant epidemiological data (10.2%) and in accessing reference isolates or DNA collections (10.2%) were also frequently reported. Notably, nearly one-third of respondents (31.6%) reported facing a combination of these challenges, while 11.7% reported other relevant difficulties. The underlying causes of these obstacles were attributed primarily to limited access to advanced testing methods (25.5%) and the lack of standardised diagnostic protocols (19.1%). Additional contributing factors included inadequate laboratory infrastructure (6.4%) and ongoing uncertainty regarding the clinical significance of *Blastocystis* (6.4%). Other issues, collectively reported by 42.6% of respondents, included challenges in obtaining ethical approval for research and the exclusion of *Blastocystis* from routine molecular diagnostics.

When questioned whether NGS could represent a cost-effective option for real-time *Blastocystis* subtype profiling, respondents expressed mixed views. The largest proportion (43.7%) was uncertain, while 37.7% considered NGS cost-effective and only 18.6% disagreed. Additional comments reflected this nuanced perspective: many respondents noted that NGS remains expensive (40.9%); however, a substantial proportion (36.4%) felt it should be accessible when required, and others emphasised that further methodological development is needed to optimise its utility for subtyping and profiling (22.7%).

Respondents also showed considerable interest in collaborative initiatives. More than half (57.8%) expressed willingness to participate in networks for sharing biological samples, sequence data, and related datasets, viewing such collaboration as an important means of enhancing data sharing, validating methodologies, improving research efficiency, and fostering joint publications. By contrast, 42.2% were not interested, mainly because they do not routinely use molecular techniques for *Blastocystis* diagnosis, analyse only small numbers of samples, or encounter *Blastocystis* infrequently. Similarly, 57.9% of respondents expressed interest in contributing to a web-based typing platform for archiving and comparing *Blastocystis* subtypes, recognising the relevance of metadata sharing; the remaining 42.1% cited the absence of subtyping or sequencing activities in their laboratories, or limited expertise, as the main reasons for not participating.

### 3.4 Awareness and perceived clinical relevance of *Blastocystis*

A total of 158 valid questionnaires were analysed from respondents who identified themselves as clinicians. Most were familiar with *Blastocystis* (94.9%) and correctly recognised it as an intestinal microorganism (88.6%). Nearly all respondents (97.4%) were aware that *Blastocystis* can be detected in both symptomatic and asymptomatic individuals, and the majority (85.5%) reported involvement in research on *Blastocystis* in humans.

When asked whether *Blastocystis* is routinely included in parasitological stool examination reports at their institution/hospital, just over half of respondents (53.2%) answered affirmatively, while 34.8% reported that it was not routinely included, and 12.0% were unsure. Among those whose laboratories routinely reported *Blastocystis*, 40.5% indicated that reporting was independent of organism burden, whereas 25.0% stated that reporting depended on detecting at least five *Blastocystis* cells/stages per microscopic field; notably, 34.5% were unsure of the specific reporting criteria used at their institution.

In cases of suspected gastrointestinal parasitic infection, most clinicians (68.4%) stated that they would examine three consecutive stool samples, processed and reported separately. Smaller proportions examined two consecutive samples (16.8%) or relied on a single stool sample (14.8%).

Almost all respondents (96.2%) considered *Blastocystis* to be linked to gastrointestinal symptoms. Among them, most (77.1%) would treat only symptomatic patients, while 15.3% would treat all *Blastocystis*-positive cases regardless of symptoms, and 7.6% would not recommend treatment. Regarding possible associations with extraintestinal symptoms such as urticaria, 64.6% of respondents believed that such a relationship exists, while only 7.0% believed that no association exists; the remaining respondents were either uncertain (22.2%) or acknowledged that such an association has been reported but remained unconvinced of its validity (6.3%). Among clinicians who believed in an association between *Blastocystis* and extraintestinal symptoms, most (73.0%) would treat only symptomatic patients, while smaller proportions would withhold treatment (14.6%) or treat all *Blastocystis*-positive patients regardless of symptoms (12.4%).

### 3.5 Treatment approaches for *Blastocystis*

Among the 158 clinicians analysed, pharmacological therapy was the predominant treatment approach for patients presenting with both gastrointestinal (61.5%) and extraintestinal (78.4%) symptoms. Probiotics/prebiotics and dietary interventions were used far less frequently: for gastrointestinal symptoms, these approaches were adopted by 8.2% and 7.3% of respondents, respectively, whereas for extraintestinal manifestations, the corresponding proportions were 12.2% and 4.1%. Combined therapeutic strategies, including herbal remedies such as garlic, ginger, and herbal teas, were also reported, particularly for gastrointestinal complaints (23.0%).

For patients with gastrointestinal symptoms, metronidazole was by far the most commonly prescribed drug (57.8%). Other frequently used regimens included trimethoprim–sulfamethoxazole (12.2%), metronidazole combined with trimethoprim–sulfamethoxazole (5.6%), and metronidazole combined with paromomycin (4.4%). Alternative drugs, including albendazole, amphotericin B, itraconazole, nitazoxanide, nitrofurans, ornidazole, secnidazole, and tinidazole, were prescribed only rarely. Table 2 compiles the drug combinations prescribed by respondents, along with their corresponding antimicrobial-resistance (AMR) potential, microbiome disruption, and reported effectiveness against *Blastocystis*.

**Table 2:** Drug therapy approaches used by the participant clinicians to treat *Blastocystis* with their corresponding pathogen target, AMR potential, microbiome disruption and reported effectiveness against *Blastocystis.*

Drug/class	Primary pathogen target	AMR potential	Microbiome disruption	Reported effectiveness against *Blastocystis* and quality of evidence (clinical trial vs. case report)
Trimethoprim-sulfamethoxazole (TMP/SMX)	Broad-spectrum Gram– and Gram+ bacteria	High (widespread resistance reported)	High (broad activity disrupts gut microbiota - dysbiosis)	22% (2/9 adults)^1^[19] 93–94% (36/38 children and 14/15 adults)^2^[20] 100% (1/1 adult)^2^[21]
Metronidazole (MZ)	Anaerobic bacteria (Gram – and Gram +); protozoa	Moderate (documented resistance in *Bacteroides, H. pylori*)	Moderate-high (targets anaerobic gut flora)	0% (0/1 adult)^2^[21] 33% (4/12 adults)^1^[19] 71% (15/21 adults)^1^[22] 100% (1/1 adult)^2^[23]
Secnidazole	Anaerobic bacteria; protozoa	Moderate	Moderate-high	60% (6/10 adults, combined with TMP/SMX)^1^[24]
Tinidazole	Anaerobic bacteria; protozoa	Moderate (similar to Metronidazole)	Moderate-high	46% (17/37 adults)^1^[25]
Ornidazole	Anaerobic bacteria; protozoa	Moderate	Moderate-high	90% (18/20 adults)^1^[22]
Itraconazole	Fungi (antifungal)	Low-moderate (emerging fungal resistance)	Low-moderate	No evidence of efficacy
Amphotericin B	Fungi (antifungal)	Low (fungal resistance rare but emerging)	Low-moderate affect fungal microbiome component)	No evidence of efficacy
Nitazoxanide	Broad-spectrum Protozoa, viruses, limited anaerobic bacteria	Low (multitarget mechanism reduces resistance risk)	Low-moderate	97–100% (NA/NA children)^1^[26] 100% (1/1)^2^[27]
Nitrofurans (e.g.*,* nitrofurantoin)	Primarily Gram – bacteria (urinary pathogens)	Low (limited resistance due to multiple mechanisms)	Low (minimal gut exposure)	No evidence of efficacy
Paromomycin	Mainly protozoa; limited Gram – bacteria	Low-moderate (less systemic exposure reduces resistance pressure)	Low (poorly absorbed; localised gut effect)	77% (40/52 adults)^1^[28] 100% (3/3 adults)^2^[29] 100% (1/1 adult)^2^[30]
Albendazole	Helminths	Not applicable	Minimal (limited direct microbiome effect)	No evidence of efficacy
Antihistamines	Histamine H1 receptors (host immune response)⁎	Not applicable	Minimal-low (limited indirect effects – immune modulation or gut motility changes)	Used to treat symptoms of urticaria, no direct effect on *Blastocystis*
Corticosteroids	Broad immunosuppressive/anti-inflammatory effects (host-targeted)⁎	Not applicable	Moderate (indirect effect – immune suppression and changes in host physiology)	No evidence of efficacy

NA, Not available.

⁎ Not a pathogen.

1 Clinical trial.

2 Case report.

Among probiotic and prebiotic options, *Saccharomyces boulardii* was the most frequently recommended (53.3%), followed by *Lactobacillus* species (20.0%). Other treatments, such as *Bacillus clausii*, *Enterococcus faecium* SF68, and combinations including *Lactobacillus* species with *Escherichia coli* Nissle 1917, were used only occasionally. Dietary recommendations for gastrointestinal symptoms most commonly included a diarrheal diet (40.0%), followed by a fibre-rich diet (26.6%) and a low-fat, low-milk diet (20.0%). Less frequently recommended approaches included reduced-fibre diets, steamed foods, and fermented products such as yoghurt or kefir.

For patients with extraintestinal symptoms, metronidazole remained the most commonly prescribed treatment (43.2%), followed by antihistamines (22.7%). Combination therapies were also used: metronidazole with trimethoprim–sulfamethoxazole (6.8%), metronidazole with antihistamines and corticosteroids (4.5%), and metronidazole with paromomycin (4.5%) (Table 2). Other treatments, such as amphotericin B, itraconazole, and paromomycin alone, were rarely prescribed. Among adjunctive therapies, *S. boulardii* and *Lactobacillus* species (50.0% each) were the most commonly cited probiotic options. Recommended dietary interventions included a diarrheal diet and a reduced-fibre diet combined with steamed foods (50.0% each).

When the analysis was restricted to clinicians reporting a single treatment modality (*n* = 94), a significant association was observed between treatment type and the criteria used to initiate it (χ^2^ = 6.411, *p* = 0.041; Fisher–Freeman–Halton exact test, *p* = 0.032). Although most clinicians in all groups limited treatment to symptomatic patients, those recommending probiotics/prebiotics alone were more likely to treat all *Blastocystis*-positive patients regardless of symptoms (40.0%) than those prescribing drugs (10.7%), whereas clinicians recommending dietary interventions (22.2%) did not differ from either group. Given the small size of the probiotic/prebiotic and diet subgroups (*n* = 10 and *n* = 9, respectively), this finding should be interpreted with caution. Most respondents (72.4%) reported conducting follow-up evaluations after treatment; among these, more than half (58.4%) assessed both clinical resolution and parasitological clearance, 33.7% monitored only symptom resolution, and 7.9% relied exclusively on confirmation of a negative diagnostic test.

## 4 Discussion

To our knowledge, this questionnaire-based study constitutes the largest multinational European survey of professionals engaged with *Blastocystis* across the human, animal, and environmental sectors, comprising 439 valid questionnaires from 38 countries and spanning diagnosis, subtyping, perceived clinical relevance, and treatment. The survey included a dedicated section to be answered by clinicians only. Based on the survey, clinicians hold a strong, broadly shared professional conviction that the organism is clinically relevant. Contrary to this, the diagnostic and molecular capacity to characterise *Blastocystis* lags substantially behind this conviction. Comparable initiatives for other intestinal protists have been narrower in scope; for example, prior European surveys of *Cryptosporidium* and *Giardia* diagnostics were largely restricted to specialist parasitology laboratories [36]. The breadth of the present dataset, therefore, offers an unusually comprehensive, if uneven, snapshot of current practice.

Across all participants (both clinicians and non-clinicians), the detection landscape remains microscopy-dominated. Microscopy was the primary screening tool for 61.6% of respondents, while only 16.6% relied solely on molecular methods. This is difficult to reconcile with the evidence: molecular assays consistently outperform microscopy in terms of sensitivity, and microscopic detection is strongly operator-dependent, varying with the examiner's experience and the morphological form present [17], [37]. Combined microscopy–molecular workflows have accordingly been recommended [37]. The persistence of microscopy as the dominant approach thus reflects a gap between what is known and what is practised; possibly driven by clinical illiteracy, cost, infrastructure, and established laboratory routine rather than by diagnostic rationale.

A parallel gap concerns subtyping. Although *Blastocystis* subtype distribution varies markedly across hosts, regions, and countries, performing subtyping is the exception rather than the rule: three-quarters of surveyed laboratories (75.0%) reported not performing it at all. Importantly, the rationale for conducting subtyping in research settings, particularly for epidemiological and population-based investigations, differs substantially from its potential role in clinical practice and should therefore be considered separately.

From an epidemiological and research perspective, an agreed upon standardised subtyping is essential for generating comparable data across laboratories, monitoring subtype distribution patterns, investigating transmission dynamics, and improving our understanding of host specificity, zoonotic potential, and population structure. However, because subtyping is rarely performed at the point of detection, most *Blastocystis* records currently generated across Europe lack the resolution required for meaningful epidemiological analyses. This is contrary to *Cryptosporidium* and *Giardia* where well-established genotyping and multilocus sequence-typing schemes have greatly enhanced epidemiological surveillance, outbreak investigations, and the identification of emerging lineages, even though they have limited direct relevance to patient management [38], [39]. In this regard, *Blastocystis* research would greatly benefit from the wider adoption of standardised subtyping methodologies.

By contrast, the role of subtyping in routine clinical practice remains uncertain. At present, there is insufficient evidence to support the routine use of *Blastocystis* subtype information to guide clinical decision-making, predict disease outcomes, or select therapeutic interventions. Whether clinical subtyping should be implemented in diagnostic laboratories, therefore, remains a separate question that depends on its demonstrated clinical utility, as well as considerations of feasibility and cost-effectiveness. The findings of the present survey do not allow these aspects to be resolved and instead highlight the need for further studies that link subtype data to robust clinical outcomes before routine clinical implementation can be justified.

Heterogeneity was evident at the very first step of the diagnostic pathway: whether a positive result is communicated to the clinician at all. Among respondents working with human samples, 20.5% never reported *Blastocystis* findings, and a further 16.7% did so only under specific circumstances, most often when the organism burden was high. However, what constitutes a “high burden” was not consistently defined, and reporting thresholds (such as the frequently cited criterion of five *Blastocystis* cells/stages per microscopic field) appear largely conventional rather than evidence-based. As a result, clinicians may receive stool reports that omit *Blastocystis* or be unaware of the reporting criteria. This blurring of the boundary between detection and clinical relevance undermines surveillance and amplifies the downstream subtyping gap and distribution, since unreported infections are never characterised further.

Regarding treatment, metronidazole was by far the most frequently prescribed agent for gastrointestinal symptoms (57.8%), yet the efficacy data compiled here (Table 2) show its performance against *Blastocystis* to be inconsistent and at times poor, with reported eradication ranging from none to a minority of treated cases across the cited series. There have also been in vitro and clinical reports of metronidazole-tolerant or -resistant isolates [40]. Additionally, a recent double-blind, placebo-controlled, randomised descriptive pilot study by Cobbucio et al. found no significant benefit of metronidazole over placebo [41] and, as such, highlights the lack of benefit of treating *Blastocystis*. Taken together, these findings suggest that the available evidence does not fully support current prescribing practices. In contrast, agents with more favourable reported efficacy (notably paromomycin and nitazoxanide) were prescribed only rarely.

This pattern suggests that prescribing is guided more by familiarity and convention than by comparative effectiveness. The predominance of metronidazole also carries implications beyond efficacy: its moderate-to-high potential to disrupt the anaerobic gut microbiota is difficult to reconcile with the principles of antimicrobial stewardship and the One Health perspective, which advocate minimising unnecessary antimicrobial exposure [42]. Considering the current evidence, treatment decisions should be guided primarily by clinical presentation rather than parasite detection alone. A cautious approach in which therapy is reserved for symptomatic individuals, after alternative causes of symptoms (e.g.*,* concomitant gastrointestinal infections) have been appropriately excluded, appears warranted. In such cases, treatment success should be assessed primarily through clinical improvement rather than parasitological clearance. Furthermore, the development of evidence-based clinical guidelines by professional societies would help promote more consistent and rational management practices across healthcare settings.

These prescribing patterns are best understood against the backdrop of a near-universal belief among clinicians in the organism's pathogenicity. To our surprise, almost all clinicians (96.2%) considered *Blastocystis* to be linked to gastrointestinal symptoms, and 64.6% endorsed an association with extraintestinal manifestations such as urticaria, convictions supported by an extensive, mostly outdated, clinical and case-report literature (e.g.*,* the case series compiled in Table 2), along with proposed virulence mechanisms, including cysteine-protease activity, degradation of secretory IgA, disruption of epithelial tight junctions, and induction of pro-inflammatory cytokines. However, this confidence contrasts with the supporting mechanistic evidence [8], [43], which has been demonstrated largely in vitro, in silico, or in murine models, frequently under conditions of chemically induced colitis, and has not been shown to operate during natural human infection [44], [45]. It was interesting to observe that most clinicians confined treatment to patients with gastrointestinal symptoms (77.1%) and extraintestinal symptoms (12.4%), with 15.3% reported treating all positive individuals regardless of symptoms. Treating asymptomatic carriage of an organism whose pathogenic role remains contested, and which is increasingly associated with a diverse, healthy gut microbiome and favourable cardiometabolic profiles [4], [10], [11], with agents that perturb the microbiota warrants caution and merits reconsideration. This highlights a clear communication gap between clinicians and scientists, potentially reflecting limited awareness of or access to emerging evidence. As our survey did not assess respondents' familiarity with the recent literature, we were unable to quantify this potential knowledge gap. This represents a limitation of the present study and should be addressed in future surveys by including questions that assess awareness of current evidence regarding the role of *Blastocystis* in health and disease. In parallel, targeted educational initiatives may help bridge this gap and promote evidence-based clinical practice. Moreover, the need to strengthen communication and knowledge exchange within the field was already identified during the development of our COST Action [15] and is further highlighted by the present findings. As such, we need to find new strategies for academics/scientists to explain their work beyond academia (see examples of science communication patterns in the CA21105 COST Action – https://blastocystis-cost.com).

Recent evidence suggests that microbiota-directed approaches, including probiotics and dietary interventions, are being increasingly explored as alternatives or adjuncts to conventional anti-*Blastocystis* therapy [46]. Interest in these strategies arises from the growing recognition that *Blastocystis* carriage is frequently associated with a diverse and stable gut microbiota and with favourable metabolic health indicators [4], [10], [11], challenging the rationale for routine antimicrobial treatment in all cases. Probiotic formulations, particularly those containing *Lactobacillus* spp. and *S. boulardii*, have shown promising effects in reducing gastrointestinal symptoms, modulating intestinal inflammation, and, in some studies, decreasing in vitro parasite burden [47], [48], although the quality of evidence remains limited and further well-designed clinical trials are needed [46]. Likewise, dietary interventions aimed at improving gut microbial diversity, especially diets rich in plant-based foods and fibre, are gaining attention as potential tools to promote gut ecosystem resilience and favourably modulate host-microbiota-*Blastocystis* interactions rather than targeting the organism directly [11].

Across regional, national, and international scales, respondents consistently recognised the value of standardised screening and subtyping protocols, yet reported their absence equally consistently. Enthusiasm for collaborative networks was substantial and explicitly tied to standardisation, with a majority expressing willingness to share samples, sequence data, and metadata. Importantly, respondents framed the principal barrier not as money but as coordination: the most frequently cited obstacle was the lack of detailed guidelines and effective communication among research groups (44.6%), ahead of limited infrastructure and funding (15.4%). The problem, in other words, is chiefly one of unified protocols and knowledge transfer rather than resources alone, a finding that provides a clear direction for intervention. For such an intervention to succeed, any collaborative network must deliberately include resource-limited participants, so that standardisation narrows rather than widens existing capacity gaps.

The use of OMICs approaches was low overall and did not differ between institutions focused on human or animal health; instead, it was concentrated in institutions working across both sectors (33.3% vs. 7.3%). This could be driven by the potential relevance of *Blastocystis* within a One Health framework, including zoonotic transmission, environmental contamination and its role as a component of the gut microbiome. However, as this subgroup was small and the underlying factors were not explored in the present survey, this observation should be considered exploratory.

Several limitations should be considered when interpreting these findings. First, the geographic distribution of responses was uneven: Türkiye, Italy, and Spain together accounted for the largest share; no responses were received from four countries, and primary veterinary care settings were underrepresented relative to health institutions and universities. Caution is therefore warranted in extrapolating to Europe as a whole; one useful check would be to test whether the survey-derived patterns align with primary epidemiological data from the better-represented countries. Second, as a self-reported survey, the study is subject to the usual reporting and selection biases, and respondents already engaged with *Blastocystis* may not be representative of routine diagnostic practice more broadly. Finally, the exclusion of multiple-selection and low-frequency responses to create independent cohorts, while improving statistical robustness, reduced the effective sample size for some comparisons. Moreover, the supplementary comparisons showed that some of the excluded respondents, particularly those with multiple affiliations, purposes or sample sources and institutions with a combined human and animal health focus, reported a broader use of molecular screening, subtyping and OMICs approaches than the pure cohorts (Supplementary Table 1); estimates derived from the pure cohorts may therefore slightly underestimate the use of these methods.

## 5 Conclusions

Based on the subset of European countries that provided valid responses, this survey reveals a field defined by a striking asymmetry: a strong, near-universal professional belief in the clinical relevance of *Blastocystis* coexists with diagnostic and molecular practices that are too heterogeneous, and too rarely subtype-resolved, to test that belief. Microscopy still predominates where molecular methods would be more informative; subtyping, central to both epidemiology and the pathogenicity question, is performed by a minority and concentrated in settings remote from clinical care; reporting criteria are inconsistent and often undefined; and prescriptions often favour metronidazole as a treatment, despite uneven efficacy and microbiome costs. Respondents themselves identified the central remedy: uniform, evidence-based guidelines and inclusive collaborative networks that prioritise knowledge transfer and close, rather than widen, capacity gaps. Delivering this standardisation, embedded in a One Health agenda that connects the human, animal, and environmental sectors, is the necessary next step towards reliable, comparable *Blastocystis* epidemiology and, ultimately, towards resolving its contested role in human health.

## List of consortium members of Working Group 1

Mapping *Blastocystis* epidemiology and diagnostics (EU-COST 21105 One Health *Blastocystis*), arranged alphabetically:

Erjona Abazaj, Matthew Adeleke, Mesut Akil, Hafidh Akkari, Rabiye Altınbaş, Yaseen MS Al-Adilee, Carolina Allievi, Safa Amairia, Lee Andersen, Kasandra Ascuña-Durand, Victoria Austin, Adnan Ayan, Elif Aydın, Bogdan Bacescu, Ross Bacchetti, Karolina Baranowicz, Nuray Bayar-Muluk, Salem Belkessa, Yusuf B. Birma, Maria Bourquia, Kubra Can-Kurt, Ilias Chaligiannis, Ondrej Cinek, Graham Clark, Burçak Cömert-Koçak, Aleksandar Cvetkovikj, Zorica Dakic, Joanna Dabrowska, Alejandro Dashti, Busra Dedecan, Gunita Deksne, Filiz Demirel, Constance Denoyelle, Omer G. Dilek, Ehab Elmahallawy, Mahmut S. Erez, Hatice Ertabaklar, Toni Eterović, Carmen Faso, Eman Fathi, Sasan Fereidouni, Maria Francia, Aldona Gajek, Ivan Galic, Maryna Galat, Alma García-Ramos, Venemin Gega, Jacinto Gomes, Zinabu Gomba, Lidia Goterris-Bonet, David González-Barrio, Giulio Grandi, Sabina Hajizada, Jolana Havlová, Gitte Hartmeyer, Carolina Hernandez-Castro, Klara Hubackova, Laura Hurtado, Jakub Hurych, Gianluca Ianiro, Ionica Iancu, Aleksandra Ignjatović, María José Irisarri-Gutierrez, Vasana Jinatham, Paula Jorge, Kateřina Jirků, Michaela Kaduková, Emmanouil Kalaitzakis, Billur Kandemir, Anu Kantele, Frank Katzer, Burak Kayabasi, Neslihan Keleştemur, Suranga Kodithuwakku, Gordana Kozoderović, Pamela C. Köster, Ozgur Koru, Karolina Kot, Dissinviel S. Kpoda, Iliya Kwoji, Marco Lalle, Vesna Lalosevic, Anna Lass, Fatgzim Latifi, Angeliki Liakata, Panagiota Ligda, Karolina Litosova, Laura Lerma-Martínez, Ana López-González-Salido, Jacob Lorenzo-Morales, Mariana Louro, Ivan Lyutakov, Jaya Makkimane, Marianna Marangi, Rubén Martín-Escolano, Daphne Mavrides, Sadiya Maxamhud, João Mesquita, Tomislav Mestrovic, Natasa Miladinovic-Tasic, Hamed Mirjalali, Jana Mitrovic, Hannah Morris, Abdullah Muftić, Sefa Mulayim, Teita Myrseli, Doaa Naguib, Jamie Newton, Paul Nguewa, Giovanna Nunziata, Suzana Otašević, Aysegul Taylan-Ozkan, Guðný R. Pálsdóttir, Mert Pekcan, John Penders, Oltiana Petri, Danijela Petrovic-Graovac, Sven Poppert, Arutchelvan Rajamanikam, Renate Ranka, Marina Randjelovic, Marina Randjelovic, Ljubica Rashikj, Lucy J. Robertson, Monika Rudzińska, Sahar Saissi, Fernando Salvador, Fakhriddin Sarzhanov, Alba Sastre-González, Pauline Scanlan, Pauline Scanlan, Essia Sebai, Marco A. Sempere-Alcocer, Serkan Sen, Ayşe Seyer, Kurtesh Sherifi, Marjana Shehu, Neslihan Sürsal-ŞimŞek, Emrah Şimşek, Ines Sifaoui, Barbara Soba, Sabiha Sensoz, Sergio Sánchez, Gregory Spanakos, Smaragda Sotiraki, Christen R. Stensvold, Chiara Stocchero, Mario Sviben, Staffan Svärd, Beata Szostakowska, Evren Tileklioğlu, Jeffrey Tomiak, Raul Y. Tito-Tadeo, Varol Tunali, Nilgün Ulutaşdemir, Alejandra Valentín, Eric Viscogliosi, Vuk Vračar, Julia Walochnik, Roland Wesolowski, Betim Xhekaj, and Hisao Yoshikawa.

## CRediT authorship contribution statement

**Eylem Akdur-Öztürk:** Writing – original draft, Methodology, Investigation. **Ana M. Figueiredo:** Writing – original draft, Methodology, Investigation. **Isabel Guadano-Procesi:** Writing – original draft, Methodology, Investigation. **Ayşe Övgü Kinay:** Investigation, Formal analysis, Data curation. **Erdoğan Malatyali:** Resources, Methodology. **Esma Kozan:** Supervision, Methodology. **Ferhat Matur:** Methodology, Data curation. **Anja Godfrey:** Resources, Project administration. **Eleni Gentekaki:** Writing – review & editing, Validation, Conceptualization. **Anastasios D. Tsaousis:** Writing – review & editing, Visualization, Supervision, Resources, Project administration, Funding acquisition, Conceptualization. **David Carmena:** Writing – review & editing, Visualization, Validation, Supervision, Project administration, Methodology, Investigation. **Funda Dogruman-Al:** Writing – review & editing, Visualization, Validation, Supervision, Project administration, Investigation, Data curation.

## Ethics approval

This study involved a voluntary, anonymous online survey of professionals. Completion and submission of the questionnaire constituted informed consent to participate, and no identifying personal data were collected. The ethical and methodological framework is described in the published study protocol [18].

## Ethics declaration

### Informed consent and patient details

Written informed consent to take part in the study and to publish the article has been obtained from all participants or their legal representatives. The privacy rights of participants have been observed.

### Studies in human

This study was performed in compliance with relevant laws, regulatory frameworks and guidelines where the research took place. This study was approved by the 10.13039/501100003356Gazi University. (Approval No. Ref. 2023–950).

## Funding

This work was supported by COST Action CA21105 “*Blastocystis* under One Health”, funded by COST (10.13039/501100000921European Cooperation in Science and Technology; www.cost.eu).

## Declaration of competing interest

The authors declare that they have no known competing financial interests or personal relationships that could have appeared to influence the work reported in this paper.

## Data Availability

The data supporting the findings of this study are available within the article and its Supplementary material. Additional anonymised data are available from the corresponding authors upon reasonable request.

## Appendix A Supplementary data

Supplementary material 1 Supplementary File 1. Full survey questionnaire used in this study and implemented via Google Forms.

Supplementary material 2 Supplementary File 2. Glossary of terms.

Supplementary material 3 Supplementary Table 1. Comparison of respondents excluded from the pure cohorts (multiple institutional affiliations, combined human and animal health focus, multiple purposes of *Blastocystis* work, multiple sample sources, or combined treatment modalities) with the corresponding pure cohorts on key variables.

Supplementary material 4 Supplementary Table 2. Frequency, percentage (%), and cumulative percentage (%) of questionnaire responses by participating country.
