# Supplementary material for: Mapping Blastocystis epidemiology and diagnostics across Europe: A One Health survey of professional practice, perceptions and unmet needs
Source: One Health. 2026 Oct 1;23:101603. doi: 10.1016/j.onehlt.2026.101603 (PMC13659901; doi:10.1016/j.onehlt.2026.101603)
Supplement: Supplementary file 1 [file mmc1.pdf]

Blastocystis under One Health (OneHealthBlastocystis) CA21105 WG1 form

Mapping *Blastocystis* epidemiology and diagnostics

Personal information collected from this survey will not be disclosed under any circumstances

* Indicates required question

1. Email *

____

PART 1: GENERAL INFORMATION

To be filled out by all participants

2. 1.1. Please write your full name

____

3. 1.2. What Institution are you part of?

____

4. 1.3. Please note down your country of work

____

5. 1.4. In what type of Institution do you work? Please choose all that apply (can be more than one)

Check all that apply.

- [ ] Health agency (Medical Institution/Hospital/Laboratory)
- [ ] University
- [ ] Research institute
- [ ] Other

6. 1.5. If you answered "Other" in the previous question, please specify

____

7. 1.6. What is the main focus of your Institution? Please choose all that apply (can be more than one)

Check all that apply.

- [ ] Human health
- [ ] Animal health
- [ ] Food, water and environmental testing
- [ ] Other

8. 1.7. If you answered "Other" in the previous question, please specify

____
____
____
____
____

9. 1.8. What is the main purpose of the *Blastocystis* work you do? (Mark one or more ovals)

Mark only one oval.

- Clinical
- Microbiological
- Epidemiological
- Other

10. 1.9. Does your laboratory conduct OMICs studies? *

Mark only one oval.

- Yes
- No

11. 1.10. If you answered "Yes" in question 1.9., please specify

12. 1.11. If you answered "Other" in question 1.8., please specify *

PART 2: *BLASTOCYSTIS* AWARENESS AND CLINICAL RELEVANCE

TO BE FILLED OUT ONLY BY CLINICIANS

13. Are you a clinician? (Clinician is mean for this question and Part 2 to be medical doctor and see/examine the patients for diagnosing of their complaints and give treatment if needed) *

Mark only one oval.

- Yes *Skip to question 14*
- No *Skip to question 37*

PART 2: *BLASTOCYSTIS* AWARENESS AND CLINICAL RELEVANCE

Clinicians only*

14. 2.1. Before we approached you with this project and questionnaire, did you know about *Blastocystis*? *

(Mark only one oval)

Mark only one oval.

- Yes
- No

15. 2.2. Did you know where *Blastocystis* reside?

(Mark only one oval)

Mark only one oval.

- Yes
- No

16. 2.3. *Blastocystis* can be found in............
(Mark only one oval)

Mark only one oval.

- only symptomatic individuals
- only asymptomatic individuals
- both symptomatic and asymptomatic individuals

17. 2.4. Is *Blastocystis* reported in the routine laboratory results of the parasitological examination of stool samples at your institution/hospital?
(Mark only one oval)

Mark only one oval.

- Yes
- No
- I do not know

18. 2.5. If you answered "Yes" to the previous question, is it number dependent or number independent?
(Mark only one oval)

Mark only one oval.

- Number dependent (5 or more in each microscopic field)
- Number independent
- I do not know

19. 2.6. When you suspect a gastrointestinal system parasitic infection in the patient, what is your diagnostic approach?
(Mark only one oval)

Mark only one oval.

- Examination of a single stool sample and report are enough
- Examination of two consecutive stool samples reported separately should be done
- Examination of three consecutive stool samples reported separately should be done

20. 2.7. What do you think about the relationship between *Blastocystis* and gastrointestinal symptoms?
(Mark only one oval)

Mark only one oval.

- It exists
- It is reported in the litereture but I do not know if it is really exists
- It does not exist
- I do not know

21. 2.8. If you answered “It exists” in the previous question, do you give treatment? *
(Mark only one oval)

Mark only one oval.

- Only to symptomatic patient
- All Blastocystis positive patients regardless of symptoms
- No

22. 2.9. If you give treatment, please select: *
(Mark ovals as needed)

Check all that apply.

- [ ] Drug
- [ ] Probiotic, prebiotic
- [ ] Herbal
- [ ] Diet

23. 2.10. If you answered “Drug” in the previous question, please clarify (If possible, include information about the active compound, the dosage, and the duration of the treatment) *

24. 2.11. If you answered “Probiotic, prebiotic” in the 2.9. question, please clarify *

25. 2.12. If you answered “Herbal” in the 2.9. question, please clarify *

26. 2.13. If you answered “Diet” in the 2.9. question, please clarify *

27. 2.14. What do you think about the relationship between *Blastocystis* and extragastrointestinal symptoms (eg., urticaria)? *
(Mark only one oval)

Mark only one oval.

- It exists
- It is reported in the litereture but I do not know if it is really exists
- It does not exist
- I do not know

28. 2.15. If you answered “It exists” in the previous question, do you give treatment?
(Mark only one oval)

Mark only one oval.

- Only to symptomatic patient
- All Blastocystis positive patients regardless of symptoms
- No

29. 2.16. If you give treatment, please select:
(Mark ovals as needed)

Mark only one oval.

- Drug
- Probiotic, prebiotic
- Herbal
- Diet

30. 2.17. If you answered “Drug” in the previous question, please clarify (If possible, include information about the active compound, the dosage, and the duration of the treatment)

31. 2.18. If you answered “Probiotic, prebiotic” in question 2.16., please clarify

32. 2.19. If you answered “Herbal” in question 2.16., please clarify

33. 2.20. If you answered “Diet” in question 2.16., please clarify

34. 2.21. After treatment do you follow up the patients?

Mark only one oval.

- Yes
- No

35. 2.22. If you answered “Yes” in the previous question:

Mark only one oval.

- Check if clinical symptoms disappear
- Check if the diagnostic test is negative
- Check both of the above

36. 2.23. Do you conduct any research on *Blastocystis* in humans?

Mark only one oval.

- Yes
- No

PART 3: *BLASTOCYSTIS* DETECTION

To be filled out by all participants

37. 3.1. What is the main sample source tested in your laboratory (can be more than one answer)?

Check all that apply.

- [ ] Human
- [ ] Animal
- [ ] Environmental

38. 3.2. What are the main methods used for screening *Blastocystis* in your laboratory? (You can choose more than one option)

Check all that apply.

- [ ] Microscopy with saline
- [ ] Microscopy with Lugol’s solution
- [ ] Microscopy with Trichrome staining
- [ ] Concentration method
- [ ] Culture
- [ ] Conventional PCR
- [ ] Real-time PCR
- [ ] Other
- [ ] None

39. 3.3. If you answered “Concentration method” in the previous question, please specify

40. 3.4. If you answered “Culture” in question 3.2., please specify

41. 3.5. If you answered “Conventional PCR” in question 3.2., please mention primer sets used

42. 3.6. If you answered “Real-time PCR” in question 3.2., please mention the primer and probe used

43. 3.7. If you answered “Other” in question 3.2., please specify

44. 3.8. What are the main methods used for subtyping *Blastocystis* in your lab? (You can choose more than one option)

Check all that apply.

- [ ] Sequenced tagged site primers (STS-PCR)
- [ ] Barcoding and DNA sequencing
- [ ] Next Generation Sequencing
- [ ] Other
- [ ] No subtyping

45. 3.9. If you answered "Other" in the previous question, please specify

46. 3.10. If you answered "Next Generation Sequencing " in the 3.8. question, please specify the platform used *

47. 3.11. If you conduct diagnosis of *Blastocystis* sp. in humans, please provide an estimate of the number of samples analysed per year
(Mark only one oval)

Mark only one oval.

- ◯ >5000 samples
- ◯ 1000-5000 samples
- ◯ 500-999 samples
- ◯ 100-499 samples
- ◯ <100 samples

48. 3.12. If you conduct diagnosis of *Blastocystis* sp. in humans, do you report the findings to a clinician?
(Mark only one oval)

Mark only one oval.

- Always
- Never
- Sometimes

49. 3.13. If you answered “Sometimes” in the previous question, please specify

50. 3.14. If you conduct diagnosis of *Blastocystis* sp. in animals, please provide an estimate of the number of samples analysed per year
(Mark only one oval)

Mark only one oval.

- >5000 samples
- 1000-5000 samples
- 500-999 samples
- 100-499 samples
- <100 samples

51. 3.15. If you conduct diagnosis of *Blastocystis* sp. in environmental (water, soil, fresh produce) samples, please provide an estimate of the number of samples analysed per year
(Mark only one oval)

Mark only one oval.

- ◯ >1000 samples
- ◯ 500-999 samples
- ◯ 100-499 samples
- ◯ <100 samples

52. 3.16. What is the preferred method to preserve stool samples for molecular testing procedures in your lab (can be more than one answer)?

Mark only one oval.

- ◯ None. We only use fresh faecal material
- ◯ Refrigeration at 4 °C
- ◯ Freezing at –20 °C
- ◯ Freezing at –80 °C
- ◯ 70% ethanol
- ◯ Commercial storage device
- ◯ Other
- ◯ We do not use molecular testing

53. 3.17. If you answered “Commercial storage device” in the previous question, please specify

____

____

____

____

____

54. 3.18. If you answered “Other” in question 3.16, please specify

55. 3.19. Which of the following is used for DNA extraction and purification in your lab (can be more than one answer)?

Mark only one oval.

- None. We do not conduct molecular testing
- Commercial kit
- In-house method
- Faeces or cultured supernatant

56. 3.20. If you answered “Commercial kit” in the previous question, please specify the provider and kit

57. 3.21. If you answered “In-house method” in question 3.19., please specify

58. 3.22. If you answered “Faeces or cultured supernatant” in question 3.19., what is the amount of starting material that you use for DNA extraction/purification?

59. 3.23. Which of the following is used for DNA sequence analysis in your lab (can be more than one answer)?

Check all that apply.

- [ ] None
- [ ] Genbank
- [ ] PubMLST
- [ ] London School of Hygiene & Tropical Medicine (http://entamoeba.lshtm.ac.uk/ref.blasto.txt)

60. 3.24. Our project aims to standardise the screening and subtyping methods across Europe. What impact would the avaliabilty standardised methods have on *Blastocystis* diagnosis, surveillance and investigations at a regional level? Please comment

61. 3.25. What impact do you think a standardised screening and subtyping might have for *Blastocystis* diagnosis, surveillance and investigations at a national level? Please comment

62. 3.26. What impact do you think a standardised screening and subtyping might have for *Blastocystis* diagnosis, surveillance and investigations at an international level? Please comment

63. 3.27. What do you think is currently missing for the development of standard methods for screening and subtyping *Blastocystis* for surveillance and investigations? Please comment.

64. 3.28. For your screening and subtyping work, do you have difficulty in any of the following:
(can be more than one answer)

Check all that apply.

- [ ] In accessing test samples
- [ ] In selecting the most suitable test for diagnostic/subtyping purposes
- [ ] In accessing to associated epidemiological data
- [ ] In accessing isolate or DNA collections for reference purposes
- [ ] Any other relevant difficulties

65. 3.29. If you chose any of the option(s) in the previous question, please comment further

____
____
____
____
____

66. 3.30. Do you think next generation sequencing will be a cost-effective option for subtypes profiling in real time?

Mark only one oval.

- ( ) Yes
- ( ) No
- ( ) Don't know

67. 3.31. Add a comment to the previous question if you wish

____
____
____
____
____

68. 3.32. Would you participate in a collaborative network to share biological samples and sequences together with associated datasets?

Mark only one oval.

- Yes
- No

69. 3.33. Please comment on the previous question

70. 3.34. Would you contribute to the establishment of a web-based typing platform for the archiving and comparison of subtypes?

Mark only one oval.

- Yes
- No

71. 3.35. Please comment on the previous question

72. 3.36. That's it! If there is anything else you would like to add, please do so here.

This content is neither created nor endorsed by Google.

Google Forms
