# Supplementary material for: Mapping Blastocystis epidemiology and diagnostics across Europe: A One Health survey of professional practice, perceptions and unmet needs
Source: One Health. 2026 Oct 1;23:101603. doi: 10.1016/j.onehlt.2026.101603 (PMC13659901; doi:10.1016/j.onehlt.2026.101603)
Supplement: Supplementary file 2 [file mmc2.docx]

**Mapping *Blastocystis* epidemiology and diagnostics across Europe: A One Health survey of professional practice, perceptions and unmet needs**

**Supplementary File 1**

Glossary of terms.

**Clinical scope.** Related to the detection and identification of *Blastocystis* colonisation/infection, assessment of associated symptoms, therapeutic decision-making, and patient follow-up.

**Epidemiological.** Related to the distribution (prevalence), determinants (risk factors), patterns (transmission routes), and population-level health impacts of *Blastocystis* colonisation/infection.

**Microbiological.** Related to the study of the biology, physiology, and interactions of *Blastocystis*, including cultivation, morphology, and interactions with other intestinal microorganisms.

**Genomics.** The comprehensive study of the *Blastocystis* genome, including its structure, function, variation, and evolution. Encompasses characterising genetic diversity, identifying subtypes, and investigating evolutionary relationships.

**Metagenomics.** The study of *Blastocystis* genetic material recovered directly from clinical or environmental samples without prior cultivation, enabling simultaneous analysis of all microbial genomes present in a sample.

**Microbiome.** The entire community of microorganisms (bacteria, archaea, fungi, viruses, and protists) and their collective genetic material inhabiting a particular environment, such as the intestinal tract.

**Transcriptomics.** The large-scale study of all RNA molecules expressed by a cell, tissue, or organism at a given time, providing insight into gene activity and functional responses.

**Probiotics.** Live microorganisms that, when administered in adequate amounts, confer a health benefit on the host (e.g., *Lactobacillus*, Bifidobacterium, and the yeast Saccharomyces).

**Prebiotics.** Substrates selectively used by beneficial host microorganisms, conferring a health benefit; typically, non-digestible dietary fibres.
