# Supplementary material for: Mapping Blastocystis epidemiology and diagnostics across Europe: A One Health survey of professional practice, perceptions and unmet needs
Source: One Health. 2026 Oct 1;23:101603. doi: 10.1016/j.onehlt.2026.101603 (PMC13659901; doi:10.1016/j.onehlt.2026.101603)
Supplement: Supplementary file 3 [file mmc3.docx]

**Supplementary Table 1. Comparison of respondents excluded from the pure cohorts (multiple institutional affiliations, combined human and animal health focus, multiple purposes of *Blastocystis* work, multiple sample sources, or combined treatment modalities) with the corresponding pure cohorts on key variables.**

| **Key variable (questionnaire item)** | **Pure cohort (n)** | **Excluded group (n)** | **χ² (df), p** | **Categories differing significantly: pure cohort vs. excluded group (Fisher’s exact p)** |
| --- | --- | --- | --- | --- |
| **A. Multiple institution types vs. pure cohort (university or health institution only)** | | | | |
| Main purpose of *Blastocystis* work (Q1.8)† | 316 | 74 | 6.648 (3), 0.084 | Clinical: 42.4% vs. 58.1% (0.019) |
| Screening methods (Q3.2)† | 316 | 71 | 14.959 (8), 0.060 | Real-time PCR: 19.6% vs. 31.0% (0.040) |
| Subtyping methods (Q3.8)† | 301 | 69 | 1.869 (4), 0.760 | None |
| Stool preservation for molecular testing (Q3.16)† | 254 | 63 | 5.070 (7), 0.651 | None |
| Difficulties in screening/subtyping (Q3.28)† | 159 | 39 | 1.885 (5), 0.865 | None |
| Reporting findings to clinicians (Q3.12) | 239 | 60 | 4.468 (2), 0.107 | Never reported: 21.8% vs. 10.0% (0.044) |
| **B. Combined human and animal health focus vs. pure cohort (human or animal health focus)** | | | | |
| OMICs studies (Q1.9) | 369 | 36 | 25.511 (1), < 0.001‡ | Conducting OMICs studies: 7.3% vs. 33.3% (< 0.001) |
| **C. Multiple purposes of *Blastocystis* work vs. pure cohort (clinical, epidemiological or microbiological only)** | | | | |
| Screening methods (Q3.2)† | 337 | 50 | 32.923 (8), < 0.001 | Trichrome staining: 9.8% vs. 22.0% (0.017); concentration method: 30.9% vs. 46.0% (0.037); culture: 10.7% vs. 22.0% (0.034) |
| Subtyping methods (Q3.8)† | 322 | 50 | 33.546 (4), < 0.001‡ | Barcoding/Sanger sequencing: 10.6% vs. 34.0% (< 0.001); no subtyping: 77.3% vs. 56.0% (0.003) |
| **D. Multiple sample sources vs. pure cohort (human or animal samples only)** | | | | |
| Screening methods (Q3.2)† | 372 | 35 | 19.964 (8), 0.010‡ | Culture: 10.5% vs. 22.9% (0.047); conventional PCR: 12.4% vs. 34.3% (0.001) |
| **E. Combined treatment modalities vs. pure cohort (drug, probiotic/prebiotic or diet only)** | | | | |
| Criteria for initiating treatment (Q2.8) | 94 | 27 | 1.780 (1), 0.182‡ | None (treating all *Blastocystis*-positive patients: 14.9% vs. 25.9%; Fisher’s exact p = 0.247) |

Percentages refer to the proportion of respondents in each group selecting the category. Respondents with “NA” responses and low-frequency “Other” categories were excluded from each comparison. Pairwise differences were assessed by comparisons of column proportions with Bonferroni correction (p < 0.05) and confirmed with Fisher’s exact test (p values in parentheses). Questionnaire items refer to Supplementary File 1.

† Multiple-response variable: a respondent could select more than one category; the omnibus χ² is therefore reported as a descriptive summary of the overall association, and inference is based on the category-level comparisons.

‡ More than 20% of cells had expected counts below five.
