# Supplementary material for: Mapping Blastocystis epidemiology and diagnostics across Europe: A One Health survey of professional practice, perceptions and unmet needs
Source: One Health. 2026 Oct 1;23:101603. doi: 10.1016/j.onehlt.2026.101603 (PMC13659901; doi:10.1016/j.onehlt.2026.101603)
Supplement: Supplementary file 4 [file mmc4.docx]

**Mapping *Blastocystis* epidemiology and diagnostics across Europe: A One Health survey of professional practice, perceptions and unmet needs**

**Supplementary Table 2**

Frequency, percentage (%), and cumulative percentage (%) of questionnaire responses by participating country

| **Countries** | **Frequency** | **Percentage (%)** | **Cumulative Percentage (%)** |
| --- | --- | --- | --- |
| Türkiye | 101 | 23.0 | 23.0 |
| Italy | 75 | 17.0 | 40.1 |
| Spain | 60 | 13.7 | 53.8 |
| France | 27 | 6.2 | 59.9 |
| Romania | 21 | 4.8 | 64.7 |
| Greece | 14 | 3.2 | 67.9 |
| United Kingdom | 13 | 3.0 | 70.8 |
| Albania | 12 | 2.7 | 73.6 |
| Portugal | 11 | 2.5 | 76.1 |
| Austria | 10 | 2.3 | 78.4 |
| Czechia | 8 | 1.8 | 80.2 |
| Bosnia and Herzegovina | 7 | 1.6 | 81.8 |
| Croatia | 7 | 1.6 | 83.4 |
| Serbia | 7 | 1.6 | 85.0 |
| Germany | 6 | 1.4 | 86.3 |
| Belgium | 5 | 1.1 | 87.5 |
| Norway | 5 | 1.1 | 88.6 |
| Poland | 5 | 1.1 | 89.7 |
| Bulgaria | 4 | 0.9 | 90.7 |
| Lithuania | 4 | 0.9 | 91.6 |
| Denmark | 3 | 0.7 | 92.3 |
| Malta | 3 | 0.7 | 92.9 |
| The Netherlands | 3 | 0.7 | 93.6 |
| Ukraine | 3 | 0.7 | 94.3 |
| Cyprus | 2 | 0.5 | 94.8 |
| Estonia | 2 | 0.5 | 95.2 |
| Finland | 2 | 0.5 | 95.7 |
| Hungary | 2 | 0.5 | 96.1 |
| Ireland | 2 | 0.5 | 96.6 |
| Kosovo | 2 | 0.5 | 97.0 |
| Latvia | 2 | 0.5 | 97.5 |
| The Republic of North Macedonia | 2 | 0.5 | 97.9 |
| Slovenia | 2 | 0.5 | 98.4 |
| Sweden | 2 | 0.5 | 98.9 |
| Switzerland | 2 | 0.5 | 99.3 |
| Iceland | 1 | 0.2 | 99.5 |
| Montenegro | 1 | 0.2 | 99.8 |
| Slovakia | 1 | 0.2 | 100.0 |
| Total | 439 | 100.0 |  |
